# Apothecaries' Assistants and Pharmacists

**Published:** 1919-08-16

**Authors:** 


### Apothecaries' Assistants and Pharmacists.

AN OLD FEUD ENDED.

AT a largely attended meeting of members of the Pharmaceutical Society of Great Britain, held in the Central Hall, Westminster, on August 6, it was decided, by a majority vote, to make a by-law under Section 4 (*b*) of the Poisons and Pharmacy Act, 1908, to admit certain holders of the "assistants'" certificate of the Society of Apothecaries who have been in charge of certain hospital dispensaries under named conditions to the Register of Chemists and Druggists without examination.

Opinion amongst pharmacists generally has been to resist the admission to the Register of unexamined persons, but under pressure from Government Departments a compromise was arrived at, and the by-law is to be considered as a discharge of the obligations cast on the Pharmaceutical Council by the 1908 Act.

Mr. Herbert Skinner, pharmacist at the G.N.C. Hospital, who is a member of the Pharmaceutical Council, disagrees with his colleagues' action, and, with three other Councillors, formed an opposition minority to defeat the passing of the by-law. They pointed out that to obtain the Apothecaries' Hall examination only six months' pupilage was required in contrast to three years' curriculum demanded for the qualification of chemist and druggist (pharmacist). That the view of the minority opposition was held by many members is shown by the fact that the by-law was passed by a majority of 200 only.

